# Scope of Nursing Practice, Organizational Commitment, and Work Environment Following the 2023 National Regulatory Update: A Cross-Sectional Study in Makkah Cluster Hospitals, Saudi Arabia

**DOI:** 10.3390/healthcare14193207

**Published:** 2026-09-28

**Authors:** Hanan M. Alfahmi, Ali Kerari, Naji Alqahtani, Hamza Moafa

**Affiliations:** 1 Makkah Health Cluster, Makkah 24245, Saudi Arabia; hamalfahmi@moh.gov.sa; 2 Nursing Administration and Education Department, College of Nursing, King Saud University, Riyadh 11421, Saudi Arabia; alqnaji@ksu.edu.sa; 3 Community, Psychiatric and Mental Health Nursing Department, College of Nursing, King Saud University, Riyadh 11421, Saudi Arabia; hmoafa@ksu.edu.sa

**Keywords:** scope of nursing practice, nursing management, organizational commitment, nursing work environment, Saudi Arabia, leadership

## Abstract

**Highlights:**

**What are the main findings?**

- In a survey conducted more than two years after the 2023 SCFHS Scope of Nursing and Midwifery Practice update, nurses in Makkah Cluster Hospitals reported a mean actual scope-of-practice score of 5.55 (*SD* = 0.61) on a 6-point scale, a mean organizational commitment score of 4.20 (*SD* = 0.66) on a 7-point scale, with affective commitment highest among the three subscales, and a mean nursing work environment score of 3.60 (*SD* = 1.00) on a 5-point scale.
- The nursing work environment score was positively associated with overall organizational commitment (*r* = 0.56, 95% CI 0.47–0.64, *p* < 0.001) and with each of the three commitment subscales. Because the data are cross-sectional, the findings indicate associations and do not establish direction or causality.

**What are the implications of the main findings?**

- This study describes enacted scope of practice, organizational commitment, and the nursing work environment among Saudi nurses following the 2023 SCFHS update. Because the design includes no preimplementation baseline, it cannot determine whether the update changed any of these outcomes.
- Efforts should focus on broadening nurses’ scope of practice and strengthening the nursing work environment.

**Abstract:**

Background: The 2023 Saudi Commission for Health Specialties (SCFHS) Scope of Nursing and Midwifery Practice document was Saudi Arabia’s first formal, category-differentiated scope framework. Nurses’ enacted scope of practice, organizational commitment, and work environment have not been described since its publication. This study described these three constructs among nurses at Makkah Cluster Hospitals following the 2023 SCFHS update and examined the associations among them. Methods: In this cross-sectional study, 259 nurses at Makkah Cluster Hospitals completed an anonymous online survey during May–August 2025 using three validated instruments: the Actual Scope of Nursing Practice questionnaire (ASCOP), the Organizational Commitment Questionnaire (OCQ), and the Practice Environment Scale of the Nursing Work Index (PES-NWI). Scores were reported as mean item scores. Data were analyzed using descriptive statistics, independent-samples *t*-tests, and Pearson correlations in IBM SPSS Statistics, version 29; subgroup comparisons were treated as exploratory. Results: The mean ASCOP score was 5.55 (*SD* = 0.61) of a possible 6, the mean OCQ score was 4.20 (*SD* = 0.66) of a possible 7, and the mean PES-NWI score was 3.60 (*SD* = 1.00) of a possible 5. Overall scores did not differ by demographic characteristics, and no subscale difference remained significant after Bonferroni correction (all Cohen’s *d* ≤ 0.46). The PES-NWI score was positively associated with overall organizational commitment (*r* = 0.56, 95% CI 0.47–0.64, *p* < 0.001). The ASCOP score was not associated with either the PES-NWI (*r* = 0.11, *p* = 0.080) or the OCQ (*r* = 0.11, *p* = 0.084). Conclusions: More favorable ratings of the work environment were associated with higher organizational commitment, whereas enacted scope of practice was unrelated to either construct. The cross-sectional design and absence of preimplementation data prevent the study from establishing change attributable to the 2023 framework or the direction of the observed associations.

## 1. Introduction

Scope of nursing practice describes the range of activities that registered nurses are legally authorized, professionally prepared, and organizationally permitted to perform. Regulatory bodies define this range with reference to education, licensure, and demonstrated competency. The breadth of this scope has direct implications for patient safety, workforce utilization, and the standing of nursing within health systems [1]. A clearly articulated and operationally supported scope allows nurses to exercise clinical judgment with greater autonomy, participate more fully in care planning and patient education, and coordinate more effectively across disciplines [2]. A poorly defined scope, in contrast, creates role ambiguity, which research consistently links to lower job satisfaction, reduced professional efficacy, and greater turnover intention [3,4].

Several instruments have been developed to quantify how nurses enact their scope in practice. The Actual Scope of Nursing Practice (ASCOP) questionnaire, developed by D’Amour and colleagues [5], is among the most widely used. It captures the self-reported frequency of performance across six dimensions: assessment and care planning, patient and family education, communication and care coordination, integration and supervision of staff, quality of care and patient safety, and knowledge updating and utilization. The Arabic adaptation of the ASCOP has been validated in the Middle East and has demonstrated strong internal consistency (Cronbach’s α = 0.93) in Saudi nursing samples [6,7]. Because the instrument quantifies the frequency and range of nursing activities, it provides a systematic basis for comparing enacted scope across settings, periods, and demographic subgroups.

For many years, Saudi Arabia’s healthcare sector lacked a formally differentiated scope of nursing practice. The Saudi Commission for Health Specialties (SCFHS) registered nurses according to educational attainment, designating diploma graduates as nursing technicians and baccalaureate graduates as nursing specialists. However, the SCFHS did not specify the practice activities or competency expectations associated with each designation [8,9]. This regulatory gap left the boundaries of nursing roles open to local interpretation, contributing to role overlap and reduced professional clarity across the sector.

In 2023, the SCFHS Nursing and Midwifery Professional Council published the first national Scope of Nursing and Midwifery Practice document. The document was developed from 2019 to 2022 through a systematic literature review, international benchmarking, and a national stakeholder workshop involving nurses, midwives, educators, and hospital administrators [10]. It defines the scope of practice for four nursing categories across eight practice domains and explicitly links each category to educational preparation and competency level. Earlier Saudi studies provide only historical reference points for this issue. Because they were conducted in different institutions using different sampling frames and instruments, they do not provide a preregulatory baseline.

The 2023 framework was designed primarily to clarify professional roles and responsibilities, and its relevance may extend beyond clinical practice. Social Exchange Theory offers a complementary explanation. When an organization defines, provides resources, and formally recognizes a nurse’s role, the nurse may interpret this support as organizational investment and reciprocate with stronger commitment [11]. Thus, organizational commitment is a useful outcome for examining the broader correlates of scope-of-practice reform, although a cross-sectional design cannot test either proposed mechanism.

Organizational commitment has been studied extensively as a determinant of nurse retention and workforce stability. Allen and Meyer’s three-component model remains the dominant framework. It distinguishes affective commitment, defined as emotional identification with the organization; continuance commitment, defined as recognition of the costs of leaving; and normative commitment, defined as a sense of obligation to remain [11]. Affective commitment is consistently the strongest predictor of intention to stay across studies, whereas continuance commitment shows a weaker and sometimes contradictory association with retention. All three dimensions tend to be higher when nurses experience a supportive work environment, feel that their professional role is recognized, and can use their scope of practice as intended [12,13]. In the Saudi nursing literature, organizational commitment has been examined primarily in relation to leadership style, work engagement, and work–life balance [14]. Al-Dossary [14] found that engagement and leadership style jointly predicted commitment among hospital nurses in Saudi Arabia.

Understanding the work environment is important because it provides the context in which both scope of practice and organizational commitment develop. The nursing work environment encompasses leadership support, nurse participation in institutional governance, nurse–physician relationships, staffing adequacy, resource availability, and the broader foundations of quality nursing care [15]. The Practice Environment Scale of the Nursing Work Index (PES-NWI), developed from the original NWI-R, operationalizes these conditions across 30 items and has been validated in U.S. and international nursing populations [16], including comparative research across European hospital settings [17,18]. Hospitals with higher practice environment scores tend to report better patient outcomes, lower nurse burnout, and stronger organizational commitment [19,20].

Social Exchange Theory (SET) provides the main theoretical rationale for this study. The theory proposes that employees tend to reciprocate organizational support with greater loyalty and stronger performance. When nurses perceive that their practice environment provides the resources, autonomy, and collaborative structures needed to enact their full scope, SET predicts a corresponding increase in commitment [21,22].

Scope of nursing practice, organizational commitment, and the work environment are interconnected constructs. A clearly defined and well-supported scope may strengthen nurses’ sense of autonomy and role fulfillment. A positive work environment may, in turn, facilitate enactment of that scope and support sustained commitment. Despite a substantial international literature on these relationships, research examining how they operate in the Saudi healthcare system since the 2023 SCFHS framework took effect remains limited, and no Saudi study has measured all three constructs in the same sample during this period.

This study had three objectives: (1) to describe the actual scope of nursing practice, organizational commitment, and the nursing work environment among nurses at Makkah Cluster Hospitals more than two years after the 2023 SCFHS update; (2) to examine whether these scores differed across demographic groups; and (3) to examine the association between the nursing work environment and organizational commitment.

**H1.**  *More favorable ratings of the practice environment are associated with higher organizational commitment, both overall and across each of the three commitment subscales.*

## 2. Materials and Methods

### 2.1. Study Design

This paper reports the quantitative phase of a larger sequential explanatory mixed-methods study. The quantitative component used a cross-sectional survey to measure the actual scope of nursing practice, organizational commitment, and the work environment among nurses employed at Makkah Cluster Hospitals. Quantitative data were collected during May–August 2025, more than two years after publication of the SCFHS Scope of Nursing and Midwifery Practice document. The study is reported in accordance with the STROBE statement for cross-sectional studies, and the completed checklist is provided as Table S1 in the Supplementary Materials.

### 2.2. Setting

The study was conducted in hospitals within the Makkah Health Cluster in Makkah City, Saudi Arabia. The cluster model brings multiple hospitals under a single administrative framework to improve operational efficiency, standardize care delivery, and facilitate governance across affiliated facilities.

### 2.3. Participants and Sampling

Nurses employed at Makkah Cluster Hospitals were recruited through convenience sampling. Eligibility required employment either full-time or part-time at a Makkah Cluster facility and at least one year of clinical experience. Nurses working exclusively in quality control or infection control were excluded because their practice profiles differ substantially from those of ward-based clinical nurses.

The principal investigator and nurse managers at participating facilities circulated the survey link. Nurse managers only forwarded the link; they did not identify, approach, or follow up with individual nurses, had no access to the response platform, and could not determine who responded. The questionnaire was anonymous, collected no identifying information, and could be discontinued at any time without consequence. These procedures were intended to protect voluntariness and confidentiality, although recruitment through managers may still have influenced participation; this possibility is noted as a limitation in Section 4.3.

The target sample size was determined through an a priori power analysis. G*Power software (version 3.1, Heinrich Heine University, Düsseldorf, Germany) was used to calculate the required sample size, with the significance level set at α = 0.05 and the anticipated population effect size set at *f*^2^ = 0.15. Based on these assumptions, the minimum required sample size was 128 and was increased to 154 to allow for nonresponse and partial completion. The final sample comprised 259 participants, exceeding the adjusted target.

### 2.4. Data Collection Procedures and Measures

Data were collected using an anonymous online questionnaire distributed through Google Forms. The questionnaire presented the demographic section first, followed by the three validated instruments in a fixed order. Responses to all items were required.

#### 2.4.1. Demographic Variables

The demographic section captured gender, marital status, age group, employment status, educational qualification, and work shift duration.

#### 2.4.2. The ASCOP

The ASCOP is a 26-item self-report instrument that measures how frequently nurses engage in activities across six practice dimensions: assessment and care planning, teaching of patients and families, communication and care coordination, integration and supervision of staff, quality of care and patient safety, and knowledge updating and utilization. Items are rated on a 6-point scale from 1 (never) to 6 (always), yielding possible summed scores of 26–156; higher scores indicate a broader and more frequently enacted scope. To place the overall and subscale scores on a common metric, all ASCOP results in this paper are reported as mean item scores on a 1–6 scale. A sample item is “Assess the patient’s health status using a systematic approach.” The instrument was developed and validated by D’Amour et al. [5] and later adapted for Arabic-speaking populations; the Arabic version achieved acceptable reliability in a Saudi study [7]. In the present sample, Cronbach’s α was 0.95 for the total scale, and the subscale values were 0.86–0.94 (assessment and care planning, 0.87; teaching of patients and families, 0.88; communication and care coordination, 0.89; integration and supervision of staff, 0.86; quality of care and patient safety, 0.94; and knowledge updating and utilization, 0.90).

The ASCOP was selected because it measures enacted scope, defined here as the activities nurses report performing. The 2023 SCFHS framework defines scope for four nursing categories across eight practice domains. These domains function as regulatory categories and do not constitute a measurement model, and no validated instrument is currently aligned with them. The six ASCOP dimensions cover direct care, patient education, coordination, supervision, safety, and knowledge-use activities within the SCFHS domains for registered nurse categories, making the instrument a practical means of describing how broadly nurses enact their role following implementation of the framework. The ASCOP does not map one-to-one onto the SCFHS domains and was not designed to assess compliance with the framework. Thus, the results describe enacted practice and should not be interpreted as a measure of adherence to the 2023 document.

#### 2.4.3. Organizational Commitment Questionnaire (OCQ)

Organizational commitment was measured using Allen and Meyer’s 24-item questionnaire [11], which yields three subscale scores: affective commitment, continuance commitment, and normative commitment, with eight items in each subscale. Items are rated on a 7-point scale from 1 (strongly disagree) to 7 (strongly agree), and negatively worded items are reverse-scored before scores are calculated. OCQ results are reported as mean item scores on a 1–7 scale. A sample affective commitment item is “I would be very happy to spend the rest of my career with this organization.” The instrument has demonstrated a Cronbach’s α of 0.88 in a study conducted in healthcare settings [23]. In the present sample, Cronbach’s α was 0.94 for the total scale, 0.89 for affective commitment, 0.92 for continuance commitment, and 0.93 for normative commitment.

#### 2.4.4. Practice Environment Scale of the Nursing Work Index (PES-NWI)

The PES-NWI assesses organizational features of the nursing work environment. The 27-item version used in this study [16,24] covers five dimensions: nurse participation in hospital affairs (8 items), nursing foundations for quality of care (9 items), nurse manager ability, leadership, and support (4 items), staffing and resource adequacy (3 items), and collegial nurse–physician relations (3 items). Items are rated from 1 (strongly disagree) to 5 (strongly agree), yielding possible summed scores of 27–135. PES-NWI results are reported as mean item scores on a 1–5 scale, with higher scores indicating a more professionally supportive environment. A sample item is “Enough staff to get the work done.” The scale has been validated in both U.S. and non-U.S. nursing samples, with Cronbach’s α values of 0.91–0.94 across its domains [16]. In the present sample, Cronbach’s α was 0.93 for the total scale, and the subscale values were 0.85–0.93 (nurse participation, 0.93; nursing foundations, 0.92; nurse manager ability, 0.92; staffing and resource adequacy, 0.85; and collegial nurse–physician relations, 0.92). Both the total score and the five subscale scores are reported. Items were assigned to subscales according to the published instrument structure and the item order used in the administered questionnaire.

### 2.5. Ethical Considerations

Ethical approval was granted by the Institutional Review Board of King Saud University (IRB Ref. No. KSU-HE-25-694; approved 13 May 2025). Additional approval was obtained from participating hospitals as required. Participation was entirely voluntary; nurses could discontinue the questionnaire at any time without consequence, and no identifying information was collected.

### 2.6. Statistical Analysis

Data were analyzed using IBM SPSS Statistics, version 29 (IBM Corp., Armonk, NY, USA). Descriptive statistics, including means, standard deviations, frequencies, and percentages, were used to characterize the sample and summarize the three study variables. All instrument scores were computed as mean item scores, with ASCOP scores ranging from 1 to 6, OCQ scores from 1 to 7, and PES-NWI scores from 1 to 5.

Cases with missing data were excluded using listwise deletion. Demographic differences in ASCOP, OCQ, and PES-NWI scores were tested using independent-samples *t*-tests at both the overall and subscale levels. Binary groupings were used for age (younger than 35 versus 35 years or older), gender, marital status (single versus married), and educational level (diploma versus bachelor’s degree or higher). Age was dichotomized at 35 years because this cutoff corresponded to the boundary between the prespecified age bands used in the questionnaire and yielded two groups of nearly equal size (*n* = 127 and *n* = 132), supporting a stable comparison.

The assumptions underlying the *t*-tests and Pearson correlations were examined before analysis. Normality was assessed using skewness and kurtosis values, which fell within the limits of ±3 for skewness and ±8 for kurtosis. The corresponding skewness and kurtosis values were −1.66 and 2.26 for the ASCOP, 0.37 and −0.32 for the OCQ, and −0.95 and 0.51 for the PES-NWI. Homogeneity of variance was assessed using Levene’s test. All assumptions for these analyses were satisfied.

Comparisons of the three overall scores across the four demographic variables constituted the primary subgroup analysis, whereas all subscale comparisons were treated as exploratory. To reduce the risk of chance findings arising from multiple tests, subscale results were interpreted using a Bonferroni-corrected alpha within each instrument, that is, 0.05/6 = 0.008 for the six ASCOP subscales and 0.05/3 = 0.017 for the three OCQ subscales. Uncorrected *p*-values are reported in the tables so that readers can apply their preferred criterion. Cohen’s *d* was calculated for each group comparison using the pooled standard deviation, with values of 0.20, 0.50, and 0.80 interpreted as small, medium, and large, respectively.

Pearson correlation coefficients were used to examine associations between the PES-NWI total score and the OCQ total and subscale scores. Ninety-five percent confidence intervals for these coefficients were derived using Fisher’s *z* transformation. Correlations between the OCQ total score and its component subscales are reported for completeness but are not interpreted substantively because the total score is calculated from those subscales. Statistical significance was set at α = 0.05, and all tests were two-tailed.

## 3. Results

### 3.1. Participant Characteristics

Of the initial sample, 16 respondents declined to participate in the online survey, and five of the remaining 264 eligible participants were excluded because of missing data or incomplete submissions. A total of 259 nurses completed the survey, yielding a response rate of 92.5%. Most participants were female (*n* = 223, 86.1%), consistent with the gender composition typical of bedside nursing in Saudi Ministry of Health facilities. Slightly more than half were aged 35 years or older (*n* = 132, 51.0%); 24.7% were aged 30–34 years, 18.1% were aged 25–29 years, and 6.2% were younger than 25 years. Most were married (*n* = 151, 58.3%) and currently employed (*n* = 253, 97.7%). The largest educational group held a nursing diploma (*n* = 177, 68.3%), followed by a Bachelor of Science in Nursing degree (*n* = 56, 21.6%), an institute-level qualification (*n* = 17, 6.6%), and a master’s degree in nursing (*n* = 9, 3.5%). Most participants worked 8-h shifts (*n* = 237, 91.5%); the remaining 22 (8.5%) worked 12-h shifts. Table 1 presents the complete demographic profile.

### 3.2. Descriptive Statistics for the Main Study Variables

ASCOP mean item scores were near the upper end of the 6-point response scale (overall *M* = 5.55, *SD* = 0.61). Assessment and Care Planning had the highest subscale mean (*M* = 5.63, *SD* = 0.59), closely followed by Teaching of Patients and Families (*M* = 5.60, *SD* = 0.64) and Knowledge Updating and Utilization (*M* = 5.59, *SD* = 0.69). Communication and Care Coordination and Quality of Care and Patient Safety each had a mean score of 5.54. Integration and Supervision of Staff had the lowest subscale mean (*M* = 5.39, *SD* = 0.84). No validated cutoff points are available for classifying ASCOP scores as low, moderate, or high; thus, the scores are described relative to the response scale rather than assigned to categories. Table 2 presents the full subscale profile.

The overall OCQ mean was 4.20 (*SD* = 0.66) on a 7-point scale, slightly above the scale midpoint of 4. Affective Commitment had the highest subscale mean (*M* = 4.45, *SD* = 0.84), followed by Normative Commitment (*M* = 4.17, *SD* = 0.75) and Continuance Commitment (*M* = 3.99, *SD* = 0.93). No established cutoff points are available for this instrument; thus, the scores are described relative to the scale midpoint rather than classified as low, moderate, or high. Table 3 presents descriptive statistics for all three subscales.

The overall PES-NWI mean was 3.60 (*SD* = 1.00) on a 5-point scale, above the scale midpoint of 3. Among the five subscales, Nursing Foundations for Quality of Care had the highest mean (*M* = 3.80, *SD* = 1.00), followed by Collegial Nurse–Physician Relations (*M* = 3.57, *SD* = 1.08), Nurse Participation in Hospital Affairs (*M* = 3.55, *SD* = 1.04), and Nurse Manager Ability, Leadership, and Support (*M* = 3.53, *SD* = 1.06). Staffing and Resource Adequacy had the lowest mean (*M* = 3.32, *SD* = 1.10). Table 4 presents the complete subscale profile.

### 3.3. Differences in Scores by Demographic Characteristics

Overall ASCOP, OCQ, and PES-NWI scores did not differ significantly by age, gender, marital status, or educational qualification, and all corresponding effect sizes were small (Cohen’s *d* ≤ 0.34). Thus, enacted scope of practice, organizational commitment, and perceptions of the work environment were broadly comparable across demographic groups. Overall score comparisons are reported in the first row of Table 5 and Table 6 and in Table 7.

Subscale analyses were exploratory and are presented in full in Table 5, Table 6 and Table 7. Five comparisons reached *p* < 0.05. Female nurses scored higher than male nurses on Assessment and Care Planning (*p* = 0.011, *d* = 0.46) and Continuance Commitment (*p* = 0.024, *d* = 0.40). Nurses aged 35 years or older scored higher than younger nurses on Assessment and Care Planning (*p* = 0.048, *d* = 0.25) and Teaching of Patients and Families (*p* = 0.032, *d* = 0.27). Married nurses scored higher than single nurses on Knowledge Updating and Utilization (*p* = 0.033, *d* = 0.28). None of these differences remained significant after Bonferroni correction within each instrument (corrected α = 0.008 for the ASCOP subscales and α = 0.017 for the OCQ subscales), and all were associated with small effect sizes. Thus, these findings should be interpreted cautiously as possible chance findings and do not provide evidence of meaningful group differences. No PES-NWI comparison reached significance, and all PES-NWI effect sizes were small (*d* ≤ 0.33).

### 3.4. Associations Across Work Environment and Organizational Commitment

The overall PES-NWI score was positively associated with the overall OCQ score (*r* = 0.56, 95% CI 0.47–0.64, *p* < 0.001), corresponding to approximately 31% shared variance. Among the commitment subscales, the PES-NWI was most strongly associated with Normative Commitment (*r* = 0.51, 95% CI 0.41–0.59), followed by Continuance Commitment (*r* = 0.43, 95% CI 0.33–0.52) and Affective Commitment (*r* = 0.39, 95% CI 0.28–0.49); all *p* < 0.001. The confidence intervals for these three coefficients overlap, so the apparent ordering of the associations should be interpreted with caution.

Correlations between the overall OCQ score and its component subscales were high (Normative Commitment, *r* = 0.87, 95% CI 0.84–0.90; Continuance Commitment, *r* = 0.77, 95% CI 0.72–0.82; Affective Commitment, *r* = 0.74, 95% CI 0.68–0.79; all *p* < 0.001). These correlations are expected because the overall score is calculated from the subscales; accordingly, they are reported for completeness and are not interpreted substantively. Table 8 presents the complete correlation matrix with confidence intervals.

## 4. Discussion

Our findings describe enacted scope of practice, organizational commitment, and the nursing work environment in a healthcare system that has recently adopted its first national definition of nursing scope. Among the relationships examined, the work environment was positively associated with nurses’ organizational commitment. The discussion below relates these findings to the existing literature and to the practical implementation of the SCFHS Scope of Nursing and Midwifery Practice framework.

The ASCOP scores in this study were higher than those reported in earlier Saudi studies. Aljohani et al. [6] reported a national mean of 4.59 among 928 nurses, and Hamadi, Aboshaiqah, and Alanazi [7] reported a comparable mean of 4.64 across nursing categories in Al-Hasa Ministry of Health hospitals. Both studies concluded that nurses across registration categories were performing essentially the same activities, a pattern they attributed to the absence of formally differentiated scope definitions. These earlier figures provide historical benchmarks and do not constitute a baseline. They were obtained from different hospitals, regions, sampling frames, and periods, and the present study collected no preimplementation data from the same institutions. Thus, the comparison describes a difference between separate samples. It cannot be interpreted as evidence of change over time and provides no evidence that the SCFHS framework has broadened how nurses enact their scope. Only a longitudinal or quasi-experimental design comparing the same institutions before and after the update could address that question.

The subscale pattern provides detail beyond that captured by the overall score. The highest-scoring dimensions, including Assessment and Care Planning, Teaching of Patients and Families, and Knowledge Updating and Utilization, are those most closely related to everyday bedside practice. Integration and Supervision of Staff had the lowest mean and the greatest variability, indicating that nurses’ involvement in supervising colleagues, orienting new staff, and contributing to unit-level professional development varied across the sample. A similar pattern has been reported in China and Iceland, where supervisory and mentorship functions tend to be concentrated within formal management roles instead of being distributed across the clinical workforce [25,26]. The SCFHS document assigns supervisory and mentorship duties to Nurse Specialists and senior categories. One possible explanation for the lower score is that job descriptions and delegation structures have not yet been aligned with the regulation. However, the study did not examine job descriptions or delegation practices and cannot evaluate this explanation. It is presented as a hypothesis for future research.

The commitment profile, with Affective Commitment having the highest mean, is consistent with findings from other Saudi nursing studies showing that nurses can remain emotionally invested in their organizations even when working conditions are difficult [14,27]. It is also consistent with the broader literature, in which affective commitment is typically the strongest predictor of intention to stay, followed by continuance and normative commitment [11]. Continuance Commitment, which reflects the perceived costs associated with leaving an organization, had the lowest mean among the three dimensions in this study. One possible interpretation is that voluntary attachment may play a greater role in nurses’ organizational commitment than the perceived costs of leaving. This interpretation is consistent with the descriptive pattern but was not tested directly because intention to stay and turnover were not measured. Future research could examine whether retention strategies that strengthen organizational attachment have different effects from strategies focused on the perceived costs of leaving.

The PES-NWI subscale profile provides further insight into the nursing work environment. Staffing and Resource Adequacy was the lowest-scoring dimension (*M* = 3.32), whereas Nursing Foundations for Quality of Care was the highest (*M* = 3.80), with leadership, collegial relations, and nurse participation falling between these values. This pattern is consistent with findings reported across Saudi Arabia and neighboring countries, where nurses tend to rate the professional and relational aspects of their work environment more favorably than the resources available to support care delivery [28].

The absence of significant demographic differences in overall scope, commitment, and work environment scores indicates that nurses across Makkah Cluster Hospitals reported broadly similar professional experiences across the demographic characteristics examined. Similar patterns have been reported in settings where institutional policies and professional standards are applied consistently [29]. The present design, however, cannot determine whether the SCFHS framework contributed to this similarity.

A small number of subscale differences reached the conventional significance threshold, but none remained significant after correction for multiple comparisons, and all were associated with small effect sizes (*d* = 0.25–0.46). These findings are reported for transparency and should not be interpreted as established group differences. If the observed patterns among female, older, and married nurses were replicated in larger samples, they would be consistent with international research linking professional experience and role maturity to aspects of nursing practice [29,30]. Based on the present data, chance remains a plausible explanation.

One practical implication arising from the overall profile concerns support for early-career nurses. Structured mentorship, continuing education, and guided professional development are approaches used to support nurses in engaging with the full range of their scope. The present study did not evaluate these interventions. Thus, this implication should be considered a direction for practice and future research rather than a finding of the present study.

The positive association between the practice environment and organizational commitment is consistent with findings from Japan and the Middle East [31,32]. The present study extends this descriptive evidence to a Saudi context in which a national scope framework has recently been introduced. Whether working conditions become increasingly important to organizational attachment as nursing roles become more clearly regulated cannot be determined from this cross-sectional design. The magnitude of the observed association should also be considered alongside its significance. A coefficient of 0.56 corresponds to approximately 31% shared variance between organizational commitment and work environment scores, leaving most of the variance unexplained. Thus, the work environment represents one substantial correlate of commitment in this sample, while other factors not measured in this study, including workload, pay, career progression, and family circumstances, may also contribute.

The work environment was more strongly correlated with Normative Commitment than with the other two commitment dimensions. However, the confidence intervals for the three coefficients overlapped. Thus, the apparent ordering of these associations remains uncertain. One possible explanation is the reciprocity premise of Social Exchange Theory, which proposes that nurses who perceive organizational support may respond with a stronger sense of obligation toward the organization [21]. The present study did not test this mechanism. It measured perceptions of the work environment and organizational commitment at a single time point and cannot establish direction. A more supportive environment may contribute to stronger commitment, more committed nurses may evaluate their environment more favorably, or a third factor may account for both. Supportive leadership, adequate staffing, and participation in decision-making have each been proposed as contributors to this relationship [33,34,35]. These conditions are amenable to organizational policy and are plausible targets for intervention studies. However, the present data cannot establish that modifying them would change organizational commitment.

### 4.1. Implications for Implementing the 2023 SCFHS Scope Framework

Publishing the 2023 SCFHS framework established a national basis for clarifying nursing roles in Saudi Arabia. Effective implementation also requires translation of the framework into organizational practice. This process may require revisions to job descriptions, performance evaluation criteria, and professional development pathways to align them with the specifications of the framework. The present study did not evaluate implementation. Thus, the considerations presented in this section are derived from the framework itself and the broader literature, with the study findings providing relevant contextual information.

Leadership, supervision, and role development may warrant particular attention because Integration and Supervision of Staff was the lowest-scoring ASCOP dimension in this sample. Clear expectations, mentorship opportunities, and structured delegation are plausible approaches to strengthening this aspect of practice, although the present study did not evaluate these strategies. The cluster-hospital model may be well positioned to support such efforts because governance is coordinated across facilities, allowing greater consistency in role implementation, performance standards, and professional development initiatives across hospitals.

### 4.2. Implications for Healthcare Leaders and Policymakers

Four actions may warrant consideration by hospital administrators. These recommendations extend beyond what the present data can establish and should be viewed as potential responses to the patterns observed. First, job descriptions could be revised to reflect the SCFHS category-specific scope distinctions, with separate expectations for the activities of nursing technicians, registered nurses, and specialists. Second, performance appraisal systems could incorporate explicit criteria for supervision, mentorship, and professional leadership so that these activities receive formal recognition and development. Third, a structured preceptorship pathway in which experienced nurses receive protected time, preparation, and formal recognition for supervising and orienting colleagues could address the dimension with the lowest score in this sample. Fourth, staffing levels and workload distribution warrant ongoing review in light of the observed association between overall work environment quality and organizational commitment.

At the policy level, the association between practice environment quality and organizational commitment documented in this study may warrant monitoring as a systemwide indicator. Repeated measurement of ASCOP, OCQ, and PES-NWI scores across Saudi healthcare clusters would allow policymakers to examine whether these indicators change over time. The present study provides a cross-sectional reference point from a single cluster and should not be interpreted as a national baseline. Systematic monitoring could provide healthcare leaders with stronger evidence to inform workforce development as national healthcare transformation continues.

### 4.3. Strengths and Limitations

This study has several limitations. The cross-sectional design prevents temporal or causal relationships from being established between the work environment and organizational commitment, and the direction of the observed association remains unknown. No preimplementation baseline was available to assess change associated with the 2023 SCFHS update. Comparisons with earlier Saudi studies involve separate samples and cannot establish change over time.

All three instruments relied on self-report and were completed by the same participants at the same time point, creating the potential for common method bias. Participants were recruited through convenience sampling within a single health cluster, limiting the generalizability of the findings to other Saudi healthcare settings. Nurses who were more engaged with their work or held more favorable views of their organization may have been more likely to respond, potentially increasing the observed mean scores. Recruitment was assisted by nurse managers. Although managers had no access to responses and no knowledge of who participated, their involvement may still have influenced participation or how freely nurses responded.

The sample was predominantly female (86.1%) and diploma-prepared (68.3%). Although this profile reflects the composition of the Ministry of Health bedside nursing workforce, the findings primarily describe a workforce with these characteristics and may not generalize to settings with different gender or qualification distributions. The numbers of male nurses (*n* = 36) and master’s-prepared nurses (*n* = 9) were too small to support confident interpretation of subgroup findings. Age was dichotomized at 35 years for group comparisons, resulting in loss of information about the underlying age distribution. Potentially relevant variables, including years of clinical experience, hospital, clinical unit, and professional position, were not collected and could not be examined, although they may be associated with both scope of practice and organizational commitment. Longitudinal and multisite studies that include these variables would address several of these limitations.

The study also has important strengths. Three validated instruments were used to measure scope of practice, organizational commitment, and the work environment, supporting comparison with findings from other studies. The study also provides timely data on these workforce outcomes following the 2023 SCFHS update and contributes to the limited literature examining nurses’ experiences within Saudi cluster-hospital settings.

## 5. Conclusions

Nurses at Makkah Cluster Hospitals reported frequent engagement across the range of activities measured by the ASCOP following the 2023 SCFHS Scope of Nursing and Midwifery Practice update. Organizational commitment scores were slightly above the scale midpoint, with Affective Commitment having the highest mean among the three dimensions, and ratings of the work environment were also above the scale midpoint. More favorable ratings of the work environment were associated with higher organizational commitment across all three commitment dimensions.

These findings describe nursing practice within one Saudi health cluster following publication of the 2023 framework. Because the study was cross-sectional and included no preimplementation data, it cannot determine whether enacted scope increased relative to the preregulatory period or whether the framework contributed to the observed patterns. The reported associations do not establish causality or direction.

Future research should examine these relationships longitudinally to determine whether changes in practice environment quality are followed by measurable changes in organizational commitment over time. Studies conducted across multiple health clusters and hospital types would help establish the extent to which these findings apply across settings. Analyses that examine enacted scope alongside the work environment and organizational commitment, including longitudinal tests of potential mediation, could provide a more complete account of the relationships that the present cross-sectional study could examine only partially.

## Figures and Tables

**Table 1:** Demographic Characteristics of Survey Participants (*n* = 259).

Variable	*n*	%
Age		
Below 25 years	16	6.2
25 to 29 years	47	18.1
30 to 34 years	64	24.7
35 years and older	132	51.0
Gender		
Male	36	13.9
Female	223	86.1
Marital Status		
Single	108	41.7
Married	151	58.3
Educational Qualification		
Diploma in Nursing	194	74.9
Bachelor in Nursing	56	21.6
Master of Nursing	9	3.5
Work Shift		
8-h shift	237	91.5
12-h shift	22	8.5

**Table 2:** Mean Scores for ASCOP Subscales and Overall Scale (*n* = 259).

Subscale	Mean	SD
Assessment and Care Planning	5.63	0.59
Teaching of Patients and Families	5.60	0.64
Communication and Care Coordination	5.54	0.68
Quality of Care and Patient Safety	5.54	0.72
Knowledge Updating and Utilization	5.59	0.69
Integration and Supervision of Staff	5.39	0.84
ASCOP Overall	5.55	0.61

Note. ASCOP, Actual Scope of Nursing Practice.

**Table 3:** Mean Scores for OCQ Subscales and Overall Scale (*n* = 259).

Subscale	Mean	SD
Affective Commitment	4.45	0.84
Normative Commitment	4.17	0.75
Continuance Commitment	3.99	0.93
OCQ Overall	4.20	0.66

Note. OCQ, Organizational Commitment Questionnaire.

**Table 4:** Mean Scores and Standard Deviations for the PES-NWI Subscales and Overall Scale (*n* = 259).

Subscale	Items	Mean	SD
Nursing Foundations for Quality of Care	9	3.80	1.00
Collegial Nurse–Physician Relations	3	3.57	1.08
Nurse Participation in Hospital Affairs	8	3.55	1.04
Nurse Manager Ability, Leadership, and Support	4	3.53	1.06
Staffing and Resource Adequacy	3	3.32	1.10
PES-NWI Overall	27	3.60	1.00

Note. PES-NWI, Practice Environment Scale of the Nursing Work Index.

**Table 5:** Demographic Differences in Mean Scores on the Actual Scope of Nursing Practice (A-ASCOP) Subscales, with Independent-Samples *t*-Test (*n* = 259).

Demographic Variable	Category	*n*	Overall ASCOP Scale	Assessment and Care Planning	Teaching of Patients and Families	Integration and Supervision of Staff	Communication and Care Coordination	Quality of Care and Patient Safety	Knowledge Updating and Utilization
Gender	Male	36	5.46 ± 0.66	5.39 ± 0.76	5.43 ± 0.79	5.51 ± 0.66	5.45 ± 0.78	5.45 ± 0.74	5.53 ± 0.75
	Female	223	5.56 ± 0.60	5.66 ± 0.55	5.63 ± 0.61	5.38 ± 0.87	5.56 ± 0.67	5.56 ± 0.71	5.60 ± 0.68
	*p*-value		0.349	0.011 *	0.081	0.362	0.375	0.408	0.563
	Cohen’s d		0.17	0.46	0.31	0.15	0.16	0.15	0.10
Age	Younger (<35 years)	127	5.49 ± 0.64	5.55 ± 0.64	5.52 ± 0.68	5.32 ± 0.86	5.49 ± 0.71	5.49 ± 0.76	5.54 ± 0.74
	Older (≥35 years)	132	5.61 ± 0.58	5.70 ± 0.54	5.69 ± 0.58	5.47 ± 0.83	5.60 ± 0.66	5.59 ± 0.67	5.64 ± 0.63
	*p*-value		0.095	0.048 *	0.032 *	0.167	0.213	0.266	0.264
	Cohen’s d		0.21	0.25	0.27	0.18	0.16	0.14	0.15
Marital Status	Single	108	5.49 ± 0.67	5.57 ± 0.63	5.52 ± 0.69	5.40 ± 0.78	5.47 ± 0.76	5.48 ± 0.73	5.48 ± 0.79
	Married	151	5.60 ± 0.57	5.67 ± 0.56	5.66 ± 0.59	5.39 ± 0.89	5.60 ± 0.61	5.59 ± 0.71	5.67 ± 0.59
	*p*-value		0.158	0.194	0.082	0.927	0.124	0.224	0.033 *
	Cohen’s d		0.18	0.17	0.22	0.01	0.19	0.15	0.28
Education	Bachelor’s Degree or Higher	56	5.60 ± 0.60	5.69 ± 0.57	5.70 ± 0.57	5.42 ± 0.80	5.63 ± 0.64	5.58 ± 0.66	5.62 ± 0.72
	Diploma or Institute Level	203	5.53 ± 0.62	5.61 ± 0.60	5.58 ± 0.65	5.39 ± 0.86	5.52 ± 0.69	5.53 ± 0.73	5.58 ± 0.68
	*p*-value		0.457	0.403	0.212	0.839	0.293	0.669	0.717
	Cohen’s d		0.11	0.13	0.19	0.04	0.16	0.07	0.06

Note. Values represent subscale mean ± SD scores on a 6-point scale (1 = never to 6 = always). ASCOP, Actual Scope of Nursing Practice. The *p*-values are derived from independent-samples *t*-tests (equal variances assumed). * *p* < 0.05 (uncorrected). Cohen’s d is the absolute standardized mean difference between the two categories listed, calculated using the pooled standard deviation. These subscale comparisons were exploratory; against the Bonferroni-corrected alpha of 0.008 (0.05/6), none of the differences were significant. The Overall ASCOP Scale column reports the corresponding comparison for the total score; none of these reached significance.

**Table 6:** Demographic Differences in Mean Scores on the OCQ Subscales, with Independent-Samples *t*-Test (*n* = 259).

Demographic Variable	Category	*n*	Overall OCQ Scale	Affective Commitment	Continuance Commitment	Normative Commitment
Gender	Male	36	4.01 ± 0.78	4.36 ± 1.04	3.67 ± 0.95	3.99 ± 0.79
	Female	223	4.23 ± 0.64	4.46 ± 0.81	4.04 ± 0.91	4.19 ± 0.74
	*p*-value		0.059	0.514	0.024 *	0.137
	Cohen’s d		0.34	0.12	0.40	0.27
Age	Younger (<35 years)	127	4.22 ± 0.65	4.46 ± 0.87	3.99 ± 0.90	4.21 ± 0.79
	Older (≥35 years)	132	4.18 ± 0.67	4.44 ± 0.82	3.99 ± 0.96	4.13 ± 0.70
	*p*-value		0.679	0.783	0.957	0.391
	Cohen’s d		0.05	0.02	0.00	0.11
Marital Status	Single	108	4.17 ± 0.71	4.37 ± 0.85	4.00 ± 0.93	4.15 ± 0.80
	Married	151	4.22 ± 0.63	4.51 ± 0.83	3.98 ± 0.93	4.18 ± 0.71
	*p*-value		0.559	0.181	0.861	0.791
	Cohen’s d		0.07	0.17	0.02	0.04
Education	Bachelor’s Degree or Higher	56	4.10 ± 0.64	4.32 ± 0.76	3.94 ± 0.97	4.03 ± 0.63
	Diploma Level	203	4.23 ± 0.67	4.49 ± 0.86	4.01 ± 0.92	4.20 ± 0.77
	*p*-value		0.175	0.198	0.616	0.124
	Cohen’s d		0.21	0.20	0.08	0.23

Note. Values represent subscale mean ± SD scores on a 7-point scale (1 = strongly disagree to 7 = strongly agree). OCQ, Organizational Commitment Questionnaire. The *p*-values are derived from independent-samples *t*-tests (equal variances assumed). * *p* < 0.05 (uncorrected). Cohen’s d is the absolute standardized mean difference between the two categories listed, calculated using the pooled standard deviation. These subscale comparisons were exploratory; against the Bonferroni-corrected alpha of 0.017 (0.05/3), none of the differences were significant. The Overall OCQ Scale column reports the corresponding comparison for the total score; none of these reached significance.

**Table 7:** Demographic Differences in Mean ± SD Scores on the PES-NWI, with Independent-Samples *t*-Test (*n* = 259).

Demographic Variable	Category	*n*	PES-NWI Mean ± SD	*p*-Value	Cohen’s *d*
Gender	Male	36	3.32 ± 1.39	0.065	0.33
	Female	223	3.65 ± 0.92		
Age	Younger (<35 years)	127	3.60 ± 1.01	0.927	0.01
	Older (≥35 years)	132	3.61 ± 1.00		
Marital Status	Single	108	3.52 ± 1.07	0.255	0.15
	Married	151	3.67 ± 0.95		
Education	Bachelor’s Degree or Higher	56	3.50 ± 1.14	0.352	0.14
	Diploma or Institute Level	203	3.64 ± 0.96		

Note. Values represent overall scale mean ± SD scores on a 5-point scale (1 = strongly disagree to 5 = strongly agree). PES-NWI, Practice Environment Scale of the Nursing Work Index. The *p*-values were derived from independent-samples *t*-tests (equal variances assumed). No comparisons reached significance (*p* < 0.05). Cohen’s *d* is the absolute standardized mean difference between the two categories listed, calculated using the pooled standard deviation; all values indicate small differences.

**Table 8:** Pearson Correlations Among the PES-NWI and OCQ Overall and Subscale Scores (*n* = 259).

Variable	1	2	3	4	5
1. OCQ Overall	1				
2. PES-NWI Overall	0.56 [0.47, 0.64]	1			
3. Normative Commitment	0.87 [0.84, 0.90]	0.51 [0.41, 0.59]	1		
4. Continuance Commitment	0.77 [0.72, 0.82]	0.43 [0.33, 0.52]	0.55 [0.46, 0.63]	1	
5. Affective Commitment	0.74 [0.68, 0.79]	0.39 [0.28, 0.49]	0.56 [0.47, 0.64]	0.23 [0.11, 0.34]	1

Note. Values in square brackets are 95% confidence intervals (*n* = 259). All correlations among the PES-NWI and OCQ scores were significant at *p* < 0.001.

## Data Availability

The datasets generated during this study are available upon reasonable request due to legal and ethical reasons. Requests should be directed to the corresponding author at alikariri@ksu.edu.sa.

## Supplementary Materials

The following supporting information can be downloaded at https://www.mdpi.com/article/10.3390/healthcare14193207/s1. Table S1: STROBE Statement checklist of items that should be included in reports of cross-sectional studies.

## Abbreviations

The following abbreviations are used in this manuscript:

ASCOP	Actual Scope of Nursing Practice
CI	Confidence Interval
IRB	Institutional Review Board
NMPC	Nursing and Midwifery Professional Council
OCQ	Organizational Commitment Questionnaire
PES-NWI	Practice Environment Scale of the Nursing Work Index
SCFHS	Saudi Commission for Health Specialties
SD	Standard Deviation
SET	Social Exchange Theory
STROBE	Strengthening the Reporting of Observational Studies in Epidemiology
