# Supplementary material for: Scope of Nursing Practice, Organizational Commitment, and Work Environment Following the 2023 National Regulatory Update: A Cross-Sectional Study in Makkah Cluster Hospitals, Saudi Arabia
Source: Healthcare (Basel). 2026 Sep 28;14(19):3207. doi: 10.3390/healthcare14193207 (PMC13665541; doi:10.3390/healthcare14193207)
Supplement: Supplementary file 1 [file healthcare-14-03207-s001.zip › healthcare-4562805-supplementary.pdf]

Table S1. STROBE Statement: checklist of items that should be included in reports of cross-sectional studies.

Manuscript: Scope of Nursing Practice, Organizational Commitment, and Work Environment Following the 2023 National Regulatory Update: A Cross-Sectional Study in Makkah Cluster Hospitals, Saudi Arabia (Healthcare, manuscript ID 4562805). Section numbers refer to the revised manuscript; page numbers should be confirmed against the final typeset proof.

	Item	Recommendation	Reported in
**Title and abstract**	1	(a) Indicate the study's design with a commonly used term in the title or the abstract.	Title; Abstract, Methods. The term "cross-sectional" appears in both.
		(b) Provide in the abstract an informative and balanced summary of what was done and what was found.	Abstract. Structured as Background, Methods, Results, Conclusions, and states the limits of what the design can establish.
**INTRODUCTION**			
**Background/rationale**	2	Explain the scientific background and rationale for the investigation being reported.	Section 1, pages 2 to 4.
**Objectives**	3	State specific objectives, including any prespecified hypotheses.	Section 1, final three paragraphs. Three numbered objectives and hypothesis H1, page 4.
**METHODS**			
**Study design**	4	Present key elements of study design early in the paper.	Section 2.1.
**Setting**	5	Describe the setting, locations, and relevant dates, including periods of recruitment, exposure, follow-up, and data collection.	Sections 2.1 and 2.2. Data collected May to August 2025 at Makkah Health Cluster hospitals.
**Participants**	6	(a) Give the eligibility criteria, and the sources and methods of selection of participants.	Section 2.3.
**Variables**	7	Clearly define all outcomes, exposures, predictors, potential confounders, and effect modifiers. Give diagnostic criteria, if applicable.	Sections 2.4.1 to 2.4.4.
**Data sources/ measurement**	8	For each variable of interest, give sources of data and details of methods of assessment (measurement). Describe comparability of assessment methods if there is more than one group.	Sections 2.4 to 2.4.4. Instrument, scoring metric, sample item, and reliability evidence given for each of the three scales.
**Bias**	9	Describe any efforts to address potential sources of bias.	Section 2.3 (anonymity, voluntariness, and the limited role of nurse managers in recruitment); Section 4.3 (selection, non-response, and common method bias).
**Study size**	10	Explain how the study size was arrived at.	Section 2.3, a priori power analysis paragraph.
**Quantitative variables**	11	Explain how quantitative variables were handled in the analyses. If applicable, describe which groupings were chosen and why.	Section 2.6. All scores reported as mean item scores; binary demographic groupings defined, with the rationale for dichotomizing age at 35 years.
**Statistical methods**	12	(a) Describe all statistical methods, including those used to control for confounding.	Section 2.6.
		(b) Describe any methods used to examine subgroups and interactions.	Section 2.6. Primary and exploratory subgroup analyses distinguished; Bonferroni-corrected alpha specified per instrument.
		(c) Explain how missing data were addressed.	Section 2.6.

		(d) If applicable, describe analytical methods taking account of sampling strategy.	Not applicable. Convenience sampling was used and no weighting or clustering adjustment was applied; this is stated as a limitation in Section 4.3.
		(e) Describe any sensitivity analyses.	Section 2.6.
**RESULTS**			
**Participants**	13	(a) Report numbers of individuals at each stage of the study.	Section 3.1.
		(b) Give reasons for non-participation at each stage.	Section 3.1.
		(c) Consider use of a flow diagram.	Not included.
**Descriptive data**	14	(a) Give characteristics of study participants and information on exposures and potential confounders.	Section 3.1 and Table 1.
		(b) Indicate number of participants with missing data for each variable of interest.	Section 2.6 and Section 3.1.
**Outcome data**	15	Report numbers of outcome events or summary measures.	Section 3.2, Tables 2, 3, and 4.
**Main results**	16	(a) Give unadjusted estimates and, if applicable, confounder-adjusted estimates and their precision (e.g., 95% confidence interval).	Sections 3.3 and 3.4, Tables 5 to 8. Correlations reported with 95% confidence intervals; group comparisons reported with Cohen's d.
		(b) Report category boundaries when continuous variables were categorized.	Section 2.6 and Tables 5 to 7.
		(c) If relevant, consider translating estimates of relative risk into absolute risk for a meaningful time period.	Not applicable.
**Other analyses**	17	Report other analyses done (e.g., analyses of subgroups and interactions, and sensitivity analyses).	Section 3.3 (exploratory subgroup analyses) and Section 3.4.
**DISCUSSION**			
**Key results**	18	Summarise key results with reference to study objectives.	Section 4, opening paragraph, and Section 5.
**Limitations**	19	Discuss limitations of the study, taking into account sources of potential bias or imprecision. Discuss both direction and magnitude of any potential bias.	Section 4.3.
**Interpretation**	20	Give a cautious overall interpretation of results considering objectives, limitations, multiplicity of analyses, results from similar studies, and other relevant evidence.	Section 4 and Section 5.
**Generalisability**	21	Discuss the generalisability (external validity) of the study results.	Section 4.3.
**OTHER INFORMATION**			
**Funding**	22	Give the source of funding and the role of the funders for the present study and, if applicable, for the original study on which the present article is based.	Funding and Conflicts of Interest statements in the back matter.

Note. An Explanation and Elaboration article discusses each checklist item and gives methodological background and published examples of transparent reporting. The STROBE checklist is best used in conjunction with that article, which is freely available on the websites of PLoS Medicine, Annals of Internal Medicine, and Epidemiology. Information on the STROBE Initiative is available at www.strobe-statement.org.
